# A small RNA, microRNA as a potential biomolecular marker to estimate post mortem interval in forensic science: a systematic review

**DOI:** 10.1007/s00414-023-03015-z

**Published:** 2023-05-31

**Authors:** Roben Suhadi Pasaribu, Elza Ibrahim Auerkari, Antonius Winoto Suhartono, Pertti Auerkari

**Affiliations:** 1 Division of Forensic Odontology, Department of Oral Biology, Faculty of Dentistry, University of Indonesia, Jakarta, Indonesia; 2 Department of Mechanical Engineering, School of Engineering, Aalto University, Helsinki, Finland

**Keywords:** microRNAs, miRNA, Biomarkers, Post-mortem changes

## Abstract

**Background:**

Post-mortem interval (PMI) is the cornerstone of the forensic field to investigate. The examination technique by seeing the changes in the body such as algor mortis, rigor mortis, and livor mortis is a traditional technique in which accuracy is influenced by many factors. A biomolecular technique that uses microRNA (miRNA) biomarkers is developing because miRNA has good stability than other RNA, so it meets the requirements to be used for PMI estimation.

**Method:**

Following the PRISMA guidelines, journals were taken from 5 databases: Scopus, Science Direct, PubMed, Embase, and Springer. The review was carried out by two people. Inclusion criteria in this review are original research, published in the last 10 years, discussing miRNA as a biomarker for PMI estimation, and free full access. While exclusion criteria are not original research and not using English.

**Result:**

Eighteen journals were reviewed in this study. The study was conducted using test animals (rats) and human samples with tissue sources taken from the liver, skeletal muscle, blood, bone, heart, skin, saliva, semen, brain, lung, vitreous humor, spleen, and kidney. miRNA expression levels after death showed different results based on miRNA target, tissue source, and others.

**Discussion:**

The results of each study are different due to the use of different types of miRNA targets and tissue sources. miRNA has great potential to estimate PMI in forensic science, but it is necessary to control the influencing factors to obtain an accurate conclusion.

## Background

Post-mortem interval (PMI) is the period between death and inspection of the deceased [1, 2]. PMI is a crucial thing when it comes to criminal, civil, and forensic science investigations [2, 3]. After death, changes occur in the corpse, called post-mortem changes (PMCs). PMCs are various physical, molecular, and biochemical changes in dead body tissues. These are irreversible and progressive over time [4, 5] Changes in the body and the factors that influence them made researchers identify PMI and make a formula for estimating the time of death [4, 5].

At the onset of death, as in the first 24 h, there are changes such as livor mortis, corpse stiffness, and algor mortis. This condition is a traditional method used to estimate PMI [4, 6, 7]. This technique is often used in the medicolegal field, but this technique still has an error rate and is influenced by various factors [1, 2, 8]. Gender, microorganisms, age, temperature, insect activity, cause of death, body structure, and humidity influence these conditions. This factor can accelerate the decay time [2, 4].

Researchers and scientists have made efforts to find new techniques to determine PMI accurately [1, 3]. An accurate estimation technique of PMI requires a parameter which shows a constant change over time after death [2]. This definition is appropriate and compatible with the degradation of nucleic acids after death [2]. Advances in molecular science have made it possible to investigate changes in protein, DNA, and RNA over time to improve the accuracy of PMI estimation [1, 2, 7, 9].

Real-time quantitative polymerase chain reaction (RT-qPCR) is a development of tools and techniques in the biomolecular field and made it possible to study the variations of the forms and types of nucleic acids such as rRNA, mRNA, and miRNA [3]. Non-coding RNA has received attention because it has the potential as a biomarker [10]. One of the non-coding RNAs is microRNA (miRNA) [11].

MicroRNA (miRNA) was introduced by Lee et al. in 1993 [12–14]. miRNAs are a part of small non-coding RNAs. miRNAs regulate post-transcriptional gene expression by controlling messenger RNA (mRNA) translation [10, 14–16]. miRNAs are 20–25 nucleotide bases in length [11, 13, 17]. miRNAs have features that are attractive for analysis in the forensic field, such as high degradability resistance, specific tissue expression when compared to mRNAs, can be multiplied in large quantities, have increased sensitivity using the latest technology [15, 18, 19], and can be extracted together with the DNA [20].

Many studies have demonstrated that miRNAs have a significant role in biological processes such as cell proliferation, differentiation, pathogenesis, apoptosis, organ formation, defense against viruses, and metabolic control [17]. One of the essential potentials of miRNA is its use in forensic pathology [18].

Several studies have shown a continued expression of miRNAs at post-mortem intervals which offer stability to degradation under extreme conditions. This reason makes them very suitable for use in the forensic field [3, 21].

Therefore, this systematic review aims to look at the development of miRNAs as a biomarker used to estimate post-mortem interval in animals and humans to be used in forensic science. In addition, we want to see the tissue targets specific to each miRNA that has been found and used.

## Methods

### Literature search technique

The review method used the Preferred Reporting Items for Systematic Reviews and Meta-Analyses (PRISMA) protocol [22, 23]. Journals are taken from 5 electronic data banks: Scopus, Embase, PubMed, Science Direct, and Springer. The journal that was used to be reviewed was published in the last 10 years. The keyword used were “microRNA or miRNA,” “Post mortem interval or PMI,” and “Forensic.” It is carried out using the BOOLEAN system so that the result is “(((microRNA) OR (miRNA)) AND ((Post Mortem Interval) OR (PMI))) AND (Forensic).” The results are then entered into Zotero to check for duplication of journals found through search engines.

### Article selection

In selecting articles or journals to be reviewed, an examination is carried out through the inclusion and exclusion criteria. The inclusion criteria were as follows: (1) journal is an original research article, (2) journal was published within the last 10 years, from January 2012 to December 2022; (3) journals discuss microRNA as a post-mortem interval marker in the forensic field; (5) journal can answer questions from PICO (Does miRNA have potential as a biomolecular marker to estimate post-mortem interval in forensics?)

The exclusion criteria were as follows: (1) journal is not original research (literature/systematic review), (2) journals did not use English, (3) journals could not be accessed in free full text, (4) journal does not provide conclusions that discuss the topic of miRNA as a biomarker for estimating PMI.

### Data synthesis

In this review, two reviewers synthesized data independently to avoid bias when conducting the review. Synthesized data include name of the first author of the journal, year of publication, location of study, sample, and the number of samples, treatment (euthanasia technique, temperature, cause of death), source of tissue used, RNA extraction technique, amplification technique, miRNA used or target, research results, conclusions, and research limitation.

## Results

### Literature search and description

**Fig. 1:**
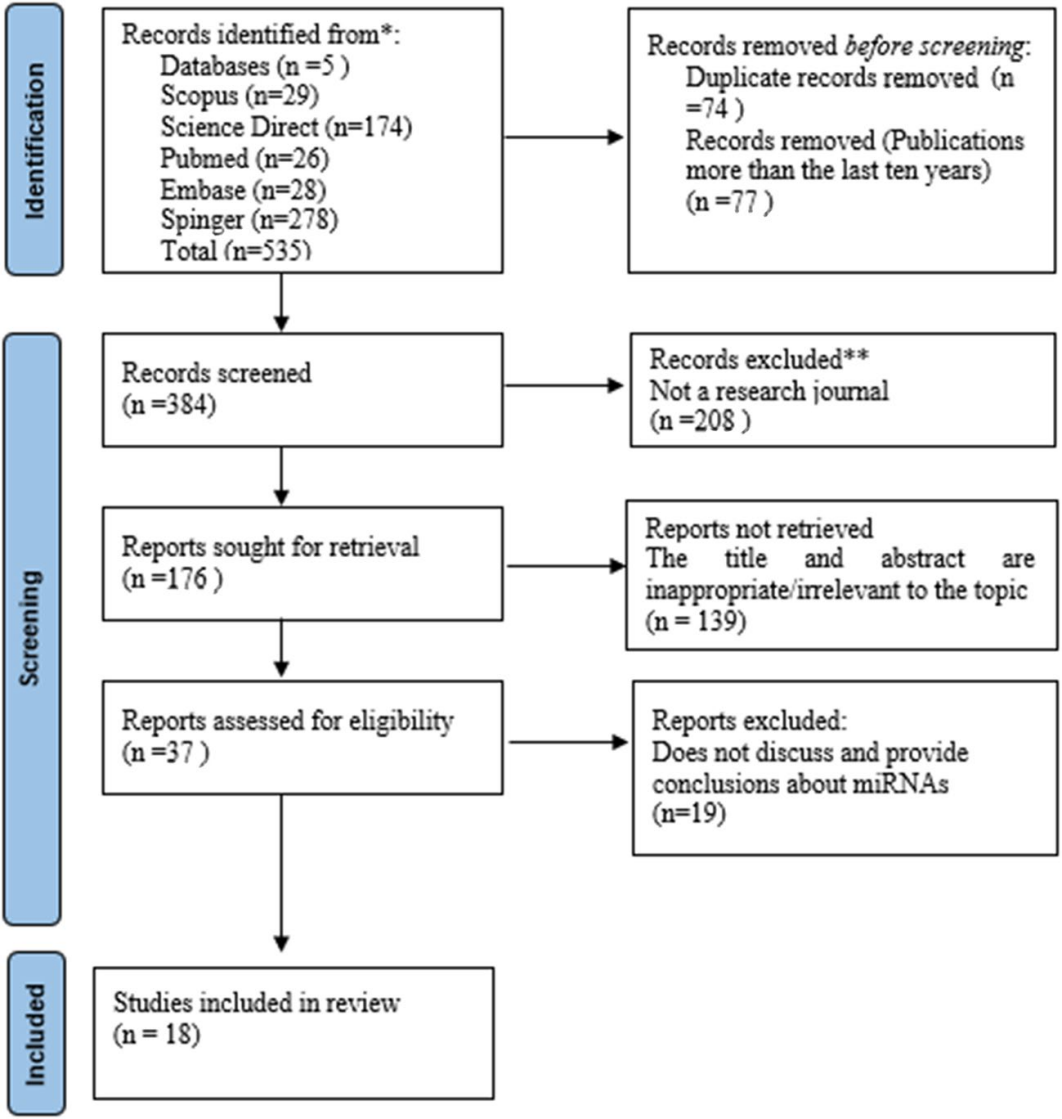
PRISMA flow chart result

The search was conducted on five electronic data banks: Scopus (“”*n* = 29), Science Direct (*n* = 174), PubMed (*n* = 26), Embase (*n* = 28), and Springer (*n* = 274) using the BOOLEAN system to obtain journals for a total number of 535. From 535 journals, 77 were automatically excluded by the system for journals published under 2012, and 74 were duplicated, bringing the total to 384. The automatic screening was carried out to exclude 208 journals that were not research journals, bringing the total journal to 176. Title and abstract screening was carried out to look for the journal that matched the inclusion criteria, as many as 139 journals, so the number of journals becomes 37. Thirty-seven journals were read entirely, and 19 journals did not meet the criteria or did not answer PICO, so the final journals that will be reviewed are 18. The PRISMA flow chart results are in Fig. 1.

### Data synthesis and results

Synthesized data include the first author’s name, year of publication, study location, sample, and the number of samples, Treatment (euthanasia technique, temperature storage, cause of death), tissue source used, RNA extraction technique, amplification technique, miRNA target, results, conclusions, and research limitations (Table 1).

**Table 1:** Journals synthesized results. D (days), H (hours), PMI (post-mortem Interval), miRNA (microRNA)

	First author	Years	Location	Sample	Treatment (euthanasia technique, temperature, time of death, cause of death)	Post-mortem Interval	Tissue source	Extraction technique	Amplification technique	miRNA target	Results	Conclusion	Research limitation
1	Derakhshanfar A et al. [4]	2022	Iran	Rats (Sprague Dawley)	Euthanasia technique using: (1) CO_2_ gas (2) ketamine/xylazine injection	2, 4, 6, 8, 10, 12, 24, and 48 H	Liver	QIAzol lysis reagent (Cat.No.79306, Qiagen, USA)	RT-qPCR	miR-122	miR-122 was significantly upregulated at 4, 10, and 24 h and downregulated at 6, 8, and 48 h for samples that were euthanized by ketamine injection compared to those treated with CO_2_ gas	Different euthanasia methods have others on the tissue and also affect the level of miR-122 expression in the liver according to the treatment	Limited in cost and research design
2	Martinez-Rivera V et al. [6]	2021	Mexico	Rats (Wistar) (*n* = 25)	Cervical dislocation	0, 3, 6, 12, and 24 H	Skeletal muscle	Trizol™ Reagent	RT-qPCR	miR-144-3p miR-23b-3p miR-381-3p	miR-114-3p decreased at 0-6 hours but not significantly miR-23b-3p significantly downregulated at 3 to 24 hours compared to controls miR-381-30 was significantly downregulated in the first 3 hours and upregulated at 6 to 24 h.	miRNAs play a role in the autolysis process. These are supported by the dysregulation of miR-23b-3p and miR-381-3p in mouse muscle at the beginning of death miR-381-3p could become a biomolecular marker to estimate post-mortem interval, but further research is needed to corroborate this marker.	The number of samples in each group is still limited.
3	Kim SY et al. [24]	2021	South Korea	Human Male (*n* = 17) Female (*n* = 11) Total (*n* = 28)	Natural death (*n* = 15) Intracerebral hemorrhagic Hypertensive, heart disease, aneurysmal subarachnoid Hemorrhagic Unnatural death (*n* = 13) Subdural Hemorrhagic drowning asphyxia	16–86 H	Blood (veins) Peripheral blood, cardiac blood, coronary sinus blood	miRNeasy serum/plasma kit (Qiagen, Hilden, Germany)	RT -qPCR	miR-16 miR-208b let-7e miR-1	miR-16 and let-7e did not show significant differences in expression levels from peripheral blood, cardiac blood, and coronary sinus blood samples miR-208 showed significantly different expression levels between the 3 sample sources. miR-1 showed a significant difference in expression levels between samples from peripheral blood and coronary sinus blood.	miR-208 and miR-1 showed significant differences in expression levels compared to blood sources. This condition did not lead to a meaningful relationship with post-mortem interval. miR-16 and let-7e had no significant relationship when associated with post-mortem interval.	Not listed
4	Na JY [19]	2020	South Korea	Human (*n* = 71)	–	< 1 months 1–3 months 3–6 months > 6 months	Bone (Patella)	TRIzol™ reagent (Thermo Fisher Scientific, Waltham, MA, 33USA)	RT-qPCR	Let-7e miR-16	The expression of let-73 and miR-16 was significantly different between the < 1-month samples compared to the other three groups.	Statistical tests showed a negative correlation between mir-16 and let-7e expressions level and post-mortem interval with correlation coefficients *R* [2] = − 0.3329 and *R* [2] = − 0.3025, respectively.	Inadequate number of samples
5	Han L et al. [26]	2020	China	Human (*n* = 71)	Sudden cardiac death (*n* = 5), mechanical asphyxia (*n* = 14), poisoning (*n* = 14), hemorrhagic shock (*n* = 14), craniocerebral injury (*n* = 14)	0–40 H	Heart (apical region)	Trizol (Invitrogen, USA)	RT-qPCR	miR-3185	miR-3185 had increased expression levels in samples that died because of mechanical asphyxia compared to other reasons.	Changes in the expression level of miR-3185 did not correlate with the time interval of death.	Not listed
6	Ibrahim SF et al. [33]	2019	Arab Saudi	Albino rats (*n* = 18)	Cervical dislocation	0, 24, and 48 H	Skin	mirVana PARIS Kit (Ambion, Austin, TX, USA)	RT-qPCR	miR-205 miR-21	miR-205 and miR-21 showed a significant increase 24 h after death, and there was a drastic decrease at 48 h	Pearson correlation test showed that the decrease in miR-205 and miR-21 expression levels did not correlate with the post-mortem interval.	The time interval for assessing the correlation of histological changes is too long.
7	Alshehhi S et al. [34]	2019	UK	Human (*n* = 19)	–	0, 7, 14, 28, 90, 180, 270, and 360 D	Saliva Semen fluid	TRI^R^ Reagent (Sigma-Aldrich, Gillingham, UK)	RT-qPCR	miR-205 miR-891a miR-10b	miR-205 in saliva showed no significant level expression decrease and remained stable over time miR-891a in semen fluids showed stable results over time from day 0–360 after death Degradation of miR-10b in semen fluids increased until day 14 and stabilized.	miRNAs have a higher stability when compared to mRNAs in the fluid of the body	The number of samples in each sample group is inadequate. The selection of specific markers for body fluids should be more comprehensive.
8	Tu C et al. [27]	2018	China	Rats BALB/cc (*n* = 45)	Cervical vertebra dislocation	0, 1, 2, 3, 4, 5, 6, 7, and 8 D	Liver Heart Skeletal muscle	Trizol reagent (Invitrogen, Carlsbad, CA, USA)	RT-qPCR	miR-122 miR133a	miR-122 and miR-133a have stabilized expression levels in Heart miR-122 has a high and sustained level of expression in the liver. miR-133a has the most stable expression in skeletal muscle	miRNAs have stability in post-mortem tissues, so selecting reference genes is necessary to serve as guidelines for estimating post-mortem interval. miR-122 and miR-133a have a stable expression that can be used as reference genes	Gene references need to be increased to 3–4, especially for the heart and liver, to improve the accuracy of post-mortem interval estimation.
9	Ye-Hui Lv et al. [9]	2017	China	Human 7 males 6 females (*n* = 13) Rats Sprague-Dawley) (*n* = 36)	5 hemorrhagic shocks 4 brain trauma 4 mechanical asphyxia Cervical dislocation Temperature 4 °C, 15 °C, 25 °C 35 °C	0,24,48,72,96,120, and 144 H	Myocardium (Apex Cordis) Liver (right lobe) Brain (frontal)	RNAiso Plus (Takara, Japan)	RT-qPCR	miR-133a miR-122 miR-1	miR-133a and miR-1 have high stability in muscle myocardium for more than five days at all temperatures. miR-122 degraded along PMI in the Liver, especially at high temperature	miR-1 and miR-133a can act as a marker or reference genes in the heart because they have high stability, while miR-122 cannot be used as a reference gene in the liver because it has degraded	Not listed
10	Ye-Hui Lv et al [28]	2016	China	Human (*n* = 12) Rats (Sprague-Dawley) (*n* = 216)	- Cervical dislocation Temperature 10 ± 1, 20 ± 1, 30 ± 1 °C	1, 3, 6, 12, 24, 36, 48, 72, 96, 120, and 144 H	Lung Muscle *(Femoral Quadriceps)* Lung	RNAiso Plus (Takara Japan)	RT-qPCR	miR-206 miR-1 miR-200c miR-195	miR-195 is stable in the Lung tissue along the post-mortem interval miR-200c is stable in the Lung tissue along the post-mortem interval miR-1 and miR-206 have stable expression levels in muscle along the post-mortem interval	miR-195 and miR-200c were chosen as reference genes in the Lung, while miR-1 and miR-206 were chosen as reference genes in muscle because they have stable characteristics	It does not display antemortem data affecting
11	Corradini B et al [35]	2015	Italy	Human (*n* = 18)	Died during the day and at night	Whole blood ≤ 48 H *Vitreous humor* ≤ 24 H	*Whole blood* *Vitreous humor*	miRNeasy Micro Kit (QIAGEN, Hilden, Germany)	RT-qPCR	miR-142-3p miR-132-3p miR-182-5p miR-26a-5p miR-96-5p miR-194-5p miR-106b-5p miR-142-5p miR-541 miR-219a-5p	miR-142-5p showed significant expression levels between samples that deceased in daytime and at nighttime miR-219 showed significant expression levels between samples that deceased in daytime and at nighttime miR-106b and miR-96 showed significant differences in expression levels between daytime and deceased nighttime samples in vitreous humor tissues	miRNAs show strong potential as they have extreme resilience after death in adverse environments and various conditions.	The number of samples is inadequate miRNA targets need to be increased
12	Yu S et al [25]	2015	South Korea	Mice (Imprinting control region) (*n* = 13)	Cervical dislocation (*n* = 3) Water drowning (*n* = 5) Salt drowning (*n* = 5)	24 H	Brain (cerebrum)	Trizol Reagent (Life Technologies Carlbas, CCA, USA)	RT-qPCR	miR-706	miR-706 showed upregulated after 24 hours in samples soaked in plain water miR-706 showed downregulated after 24 hours in samples soaked in saltwater	miR-706 showed significant differences in expression levels when given a different treatment, such as upregulated when submerged in plain water and downregulated when submerged in saltwater	Not listed
13	Ma J et al [29]	2015	China	Rats (Sprague-Dawley) (*n* = 270)	Cervical dislocation	1, 3, 6, 12, 24, 36, 48, 72, 96, 120, and 144 H	Brain	Trizol Solvent (Invitrogen, USA)	RT-qPCR	miR-125b miR-9	miR-125b has stable expression levels up to 144 h after death miR-9 has stable expression levels up to 144 hours after death	miR-125b and miR-9 have stable expression throughout the death interval, so they were used as reference genes.	Not listed
14	Ye-Hui Lv et al [3]°	2014	China	Rats (Sprague Dawley) (*n* = 12)	Cervical amputation Storage temperature 4 ± 2°C and 25 ± 2°C	0, 1, 3, 12, 24, 36, 48, 72, 96, 120, 144, 168, 192, 216, 240, 288, and 312 H	Spleen	Trizol solvent (Invitrogen, Carlsbad, CA)	RT-qPCR	miR-125b miR-143	miR-125b and miR-143 showed more stable expression along the post-mortem interval at both 25 °C and 4 °C storage temperatures.	miRNAs are an excellent choice as endogenous marker control because they are least affected by the time of death and temperature, such as miR-125b and miR-143	Not listed
15	Wen-Can L et al [31]	2014	China	Rats (Sprague Dawley)	Killed by suffocating	1, 3, 6, 12, 15, 18, 21, 24, 36, 48, 72, 96, 120, 144, and 168 H	Heart	Trizol solvent (Invitrogen, USA)	RT-qPCR	miR-1	miR-1 has a stable expression value along the postmortem interval	miR-1 is a stable miRNA. miR-1 is chosen to be an endogenous control in the heart	Not listed
16	Wang H et al [21]	2013	China	Tikus (C57) (*n* = 33)	Not listed	0.5, 1, 2, 3, 4, 6, 8, 20, 24, and 48 H	Liver	RNAiso reagent (Takara, Japan)	RT-qPCR	miR-195 miR-150 miR-122 miR-206 miR-133a	miR-206 expression levels decreased in the first 24 h after death miR-133a expression levels decreased in the first 24 h after death miR-195 expression levels rise during the first 24 h after death	Changes in expression levels occurred in all five miRNAs but did not correlate with the first 24 h of PMI	Not listed
17	Odriozola A el al [15]	2013	Spain	Human (*n* = 34)	Dying during the day Dying at night	≤ 24 H	*Vitreous humor*	miRNA Isolation Kit Ambion^R^ *mir*Vana™ (Ambion, Austin, TX)	RT-qPCR	miR-541 miR-142-5p miR-20a miR-34c miR-888 miR-671- 3p miR-484	miR-20a, miR-142-5p, miR-484, miR-541, miR-671-3p, miR-34c, and miR-888 showed significantly stable expression levels at both individual mortality times.	The miRNA expression did not correlate with the mortality interval. It is concluded that miRNAs are stable, especially 24 h after the end of life.	The number of samples is inadequate Medical data samples are incomplete, such as cause of death, previous illnesses, etc.
18	Zhangheng et al [32]	2013	China	Human (*n* = 40)	Head injury, hemorrhagic shock, mechanical asphyxia, sudden cardiac disease	< 10 H 10–20 H > 20 H	Heart Brain Kidney Skin	Trizol reagent (Invitrogen, CA)	RT-qPCR	miR-194 miR-1 miR-203 miR-9	miR-9 showed stable expression levels in the Brain at the time interval of death between 10 and 20 h in head injury cases and showed stable expression levels at the time interval of dying between 10 and 20 h in hemorrhagic shock cases miR-1 showed stable expression in the heart at the interval of death < 10 h in cases of death due to Head injury and steady at a gap of dying between 10 and 22 h in cases of mechanical asphyxia miR-194 showed stable expression levels in the Kidney at the time interval of death < 10 h, 10–20 h in cases of hemorrhagic shock and sound at the time interval of death > 20 h in cases of mechanical asphyxia miR-203 shows stable expression levels in the skin at the < 10 h mortality interval in head injury deaths	In this study, miRNA is not suitable to be used as an endogenous control.	The sample is not diverse enough. Another miRNAs target needs to be selected

From 18 journals, research conducted in several countries such as Iran (*n* = 1) [4], Mexico (*n* = 1) [6], South Korea (*n* = 3) [19, 24, 25], China (9) [9, 21, 26–32], Saudi Arabia (*n* = 1) [33], UK (*n* = 1) [34], Italy (*n* = 1) [35], Spain (*n* = 1) [15]. Of the 18 journals, nine researches used animals (rats/mice) as a sample [4, 6, 21, 25, 27, 29–31, 33]. Seven researchers used the human body as samples [15, 19, 24, 26, 32, 34, 35], research that used animals and human body as a sample in as many as two journals [9, 28].

Tissue source used in this study are varied: liver [4, 9, 27, 32], skeletal muscle [6, 27, 32], blood [24, 35], bone [19], heart [9, 21, 26, 27, 31, 32], skin [32, 33], saliva [34], semen [34], brain [21, 25, 29, 32], lungs [28], vitreous humor [15, 35], lymph [30], and kidneys [32].

The materials used to perform the RNA extraction used are different. They use QIAzol lysis Reagent (Cat. No.79306, Qiagen, USA) [4], Trizol™ reagent [6], Trizol™ reagent (ThermoFisher Scientific, Waltham, MA, USA) [19], miRNeasy serum/plasma kit (Qiagen, Hilden, Germany) [24, 35], Trizol™ reagent (Invitrogen, USA) [19, 25–27, 29–32], Tri^R^ reagent (Sigma-Aldrich, Gillingham, UK) [34], Takara, Japan, RNAiso Plus (Takara, Japan) [9, 21], Trizol reagent (Life Technologies, USA) [25], Ambion^R^ mirVana™ miRNA Isolation Kit (Ambion, Austin, TX) [15, 33]. All studies used amplification techniques to quantify miRNA expression levels by RT-qPCR.

From 18 journals, 8 journals found results in the form of changes in miRNA expression levels after an interval of death [4, 6, 15, 19, 21, 25, 26, 33]. One journal showed a negative correlation between miRNA expression levels and post-mortem interval, namely miR-16 and let-7e [19]. The results of other studies say that further research is needed for miR-3381-3p because it has great potential. In addition, 10 journals found that miRNA expression level values were stable throughout the post-mortem interval [9, 24, 27–32, 34, 35].

In addition, several different treatments were carried out. Derakhshanf et al gave different treatment when preparing the sample. They use CO_2_ gas and ketamine/xylazine injection [4]. Kim SY et al. gave different treatment by taking blood samples from different places such as peripheral blood, heart blood, and coronary sinus blood with the results significantly different miRNA expression levels [24]. Han L et al. took samples of corpses with different causes of death, like poisoning, craniocerebral injury, mechanical asphyxia, and hemorrhagic shock. miR-3185 had increased expression levels in samples that died because of mechanical asphyxia [26]. Corradini B. et al. took samples by differentiating the time of death of the samples that are during the day and at night with the results of differences in expression levels in samples that died during the day and at night [35]. Yu S et al. conducted a study by differentiating the sample immersion media, namely, plain water and salt water. Regulation of miR-706 increased in plain water, while in salt water, its regulation has decreased [25]. Lv Y et al. carried out different treatments on the samples which were stored at 25 °C ± 2 and 4 °C ± 2. The results showed that miRNA has a stable expression in both treatments [30]. Odriozola A et al. conducted different samples in the form of time of death, which are during the day and at night. The results showed stable expression levels in both the samples that died during the day and at night [15]. Zhang H et al. took samples with different causes of death, namely, head injury, hemorrhagic shock, mechanical asphyxia, and sudden cardiac disease [32].

## Discussion

PMI estimation has always been an exciting and vital challenge in forensics [36, 37]. Especially when the corpse is already severely damaged, it is more difficult to assess the lesions that show the dynamic process of death [37]. miRNAs have characteristics such as being more stable compared to longer RNAs such as mRNA [18].

Eighteen journals that discussed miRNA as one of the biomarkers to estimate post-mortem interval were reviewed. Some miRNAs decrease expression levels at the time of death increases, and some miRNAs have stable expression levels until sometime after death. All studies used RT-qPCR techniques to determine expression levels. RT-qPCR is an assay that uses fluorogenic primers to combine transfer energy by resonance fluorescence techniques with traditional RT-PCR [38, 39]. RT-qPCR is widely used because it has sensitivity and specifications in time-lapse studies of death, and RT-qPCR has reliability and accuracy in analyzing RNA levels in specimens [30].

From 18 journals, there are five journals that used the heart as a target to see miRNA expression [9, 26, 27, 31, 32]. miRNAs’s targets are miR-3185, miR-122, miR-133a, and miR-1. miR-3185 showed increasing expression levels but has no significant correlation with post-mortem interval (40H) [26]. miR-122, miR-133a, and miR-1 have stable expression throughout the post-mortem interval [9, 27]. Stability of miR-122, miR-133a, and miR-1 makes these miRNAs can be used as reference genes in the heart [9, 27]. Lv Y et al. also found that miR-1 and miR-133a in the heart showed high and stable expression levels up to 144 h [9]. Tu C et al. reported that miR-133a was also stable up to 8 days after death in cardiac and skeletal muscle [27]. Lv Y et al. found that miR-1 had stable expression levels up to 144 h after death in the femoral quadriceps muscle; this is because miR-1 has a task in the physiological formation of muscle tissue [28]. In contrast, Li We et al. found that miR-1 is stable during post-mortem intervals up to 168 h with samples from the heart [31].

Four journals used the liver as the target organ to look at the expression of miR-206, miR-150, miR-12, miR-195, and miR-133a [4, 9, 21, 27]. miR-133a and miR-206 showed a decrease in expression levels at 24 h after death while miR-195 increased in the first 24 h after death [21]. However, the expression levels of the five markers do not correlate with PMI [21].

miR-122 is a miRNA expressed in the liver and has been studied widely. Wang et al. said that miR-122 did not correlate with expression levels and PMI in the early 24 h [21]. Lv Y et al. found that miR-122 degraded in line with post-mortem interval, especially at high temperatures, but at low temperatures, it was stable until 144 h, so it was concluded that it did not meet the criteria as a reference gene [9]. In contrast, Tu C et al. research showed that miR-122 showed a stable expression level at 8 days of death in samples taken from the heart and liver [27]. Derakhshanfar A et al. said that miR-122 increased expression levels at an interval of 6, 8, and 48 h after death [4].

This condition can be different because some studies use a special treatment such as sample preparation techniques by euthanasia using CO_2_ and ketamine injection. These differences in preparation methods give different levels of expression in the liver ^4.^ Samples were stored at 4 °C, 15 °C, 25 °C, and 35 °C. There were differences in miR-122 expression levels. At 35 °C, miR-122 decreased due to faster degradation. So, it is concluded that higher temperatures will accelerate the degradation of miR-122 in the liver [9].

miR-23-3p, miR-381-3p, and miR-114-3p were tested and showed different results in skeletal muscle. miR-382-3p expression increased throughout from 0 to 24 h. In contrast, miR-23-3p showed a decrease in expression levels from 0 to 24 h, while miR-114-3p decreased at hours 0 to 6 but not significantly [6].

Kim SY et al. researched miR-1, miR-16, miR-208b, and let-7e with sample sources taken from different blood vessels. miR-1 and miR-208b demonstrated different expression levels according to tissue source, while let-7e and miR-16 expressed similar levels. However, the four markers were not significantly correlated with post-mortem intervals [24]. In contrast, Na J et al. took samples from bone, found differences in miRNA expression levels along with post-mortem interval increased, and showed a negative correlation between expression levels and PMI [19].

It was concluded that miR-16 and let-7e showed different expression results depending on the target organ/tissue. In samples taken from blood, miR-16 and let-7e did not significantly correlate between PMI and expression levels [24]. Whereas in samples taken from Pattela bone, miR-16 and let-7e showed a decrease in expression levels as the time of death increased with correlation coefficient values *R*^2^ = − 0.3329 and *R*^2^ = − 0.3025 [19].

In a study using skin samples, Zhang H et al. found miR-203 showed stable expression levels at < 10 h [32]. Meanwhile, Ibrahim SF et al. used miR-205 and miR-21 as target miRNAs in the skin. They found that miR-205 and miR-21 had increased expression levels 24 h after death and had a drastic decrease at 48 h [33]. Alshehhi S et al. found that miR-205 showed stable expression levels for 360 days after death and became one of the specific markers in saliva [34].

In human samples, the cause of death and the source/location of sampling affect the expression levels. Blood samples from different origins give different expression levels, such as peripheral blood, cardiac blood, and coronary sinus blood. For example, miR-208 showed greater expression levels in blood sourced from coronary sinus blood [24]. Deaths caused by mechanical asphyxia led to increased expression levels of miR-3185 in the heart compared to hemorrhagic shock and craniocerebral injury [26]. Odriozola et al. reported insignificant levels in miRNA expression (miR-541, miR-484, miR-34c, mir-142-5p, miR-20a, miR-888, and miR-671-3p) after death between who dies at night and during the day in vitreous humor samples [15]. Different with Corradini et al., they found significant differences in miRNA expression levels (miR-106b, miR-96, miR,142-5p, and miR-219) after death between samples that died during the day and at night in the first ≤ 24 h in the vitreous humor and ≤ 48 h in blood [35]. This difference in results could be due to differences in miRNA targets used in each study. Zhang H et al. conducted a survey on miR-1 which is considered a reference marker in the heart that showed a stable expression level in samples with a death interval of < 10 h in cases of death due to trauma, while in cases of asphyxia, it was stable in 10–22 h [32].

With this review, miRNAs are one of the biomolecular markers with excellent potential to estimate post-mortem interval. miRNA shows different expression levels when tested in other tissues, so it is necessary to choose the right miRNA target according to the target tissue. Storage of samples at different temperatures will also affect the expression level so that it can be considered during the study. The cause of death of the samples will also affect the expression level. It is necessary to conduct more in-depth research on miRNA by controlling any shortcomings in previous research studies so accurate results can be obtained using miRNA as a marker.

From this review, each study has different limitations, such as inadequate samples in each group of representatives. The target of miRNA is still limited to each tissue target. In addition, information about the condition of the sample was not obtained, such as the previous disease, the duration of the diseases. and the cause of death in some sample.

Recommendations: (1) Research is carried out by adding and selecting miRNAs specific to the target tissue. (2) The researcher must obtain all the sample’s data, including the cause of death and disease. (3) Samples are placed at room temperature to simulate corpses found in the open area. (4) Each group needs to add more sample.

## Conclusion

From this review, we can say that miRNA has enormous potential as one of the biomarkers o estimate PMI in forensic science. One reason is the stability and robustness of miRNA compared to others RNAs. Many factors affect miRNA expression levels, such as tissue source, time of death, cause of death, and temperature. Each miRNA has a target gene that will express according to its target region. The results of this review show the existence of miRNAs that have the potential as reference genes. In future research, it is necessary to pay attention to the number of adequate samples and the selection of specific miRNAs. Control of all influencing factors can reduce research bias.

## Data Availability

The reviewed data are available in the referenced publications and summarized for synthesis in Table 1.
